# COVID-19 Vaccines in Skilled Nursing Facilities: Recurring Interviews with Administrators

**DOI:** 10.1093/geroni/igab046.3677

**Published:** 2021-12-17

**Authors:** Amy Meehan, Joan Brazier, Caroline Madrigal, Renee Shield, Emily Gadbois

**Affiliations:** 1 Brown University, Providence, Rhode Island, United States; 2 Providence VA Medical Center, Providence VA Medical Center, Rhode Island, United States

## Abstract

Skilled nursing facilities (SNFs) are on the front lines of changing policies regarding the COVID-19 pandemic. The most recent development is a potential vaccine mandate for staff working in SNFs. We use ongoing findings from 130 of 160 in-depth, semi-structured interviews in progress with administrators at 40 SNFs in eight diverse healthcare markets across the United States to understand the current landscape of COVID-19 in SNFs. Four repeated interviews at 3-month intervals provide a unique longitudinal perspective on the impact of COVID-19 and SNFs’ response to vaccinations, including the vaccine mandate. Rigorous thematic analysis reveals insights into administrator responses and creative approaches to address vaccine hesitancy, and future expectations for SNF operations in light of the vaccine and the mandate. Administrators express cautious hope that the vaccine will allow SNFs to return to a new normal of daily life for residents in terms of family visitations, communal dining, and resident activities. Overriding questions include how to overcome persistent vaccine hesitancy from SNF staff who cite fear of side effects despite education initiatives and how to stem staff retirement or transition to other healthcare settings. SNFs represent a microcosm of the country’s concerns as a whole. Insight into the evolving and complex dynamics shed important light on national trends and help provide solutions for moving forward. Findings from this study have implications for policymakers and SNF leadership as they consider ways to promote vaccination and retain staff amid vaccine mandates.

